# Supplementary material for: Effectiveness of a respiratory rehabilitation program including an inspiration training device versus traditional respiratory rehabilitation: a randomized controlled trial
Source: PeerJ. 2023 Dec 15;11:e16360. doi: 10.7717/peerj.16360 (PMC10726745; doi:10.7717/peerj.16360)
Supplement: Supplemental Information 4 [file peerj-11-16360-s004.pdf]

ClinicalTrials.gov Search Results 07/23/2023

	Title	Status	Study Results	Conditions	Interventions	Locations
1	Comparison of Two Respiratory Physiotherapy Treatments in Postcovid-19 Patients.	Completed	No Results Available	• COVID-19<br>• Respiratory Distress Syndrome	• Device: PowerBreathe and PEP Therosold tools	• Universidad Católica de Ávila, Ávila, Spain
